# Interfacial structures and energetics of the strengthening precipitate phase in creep-resistant Mg-Nd-based alloys

**DOI:** 10.1038/srep40540

**Published:** 2017-01-17

**Authors:** D. Choudhuri, R. Banerjee, S. G. Srinivasan

**Affiliations:** 1 Department of Materials Science and Engineering, University of North Texas, Denton, TX, 76191, USA

## Abstract

The extraordinary creep-resistance of Mg-Nd-based alloys can be correlated to the formation of *nanoscale-platelets* of β_1_-Mg_3_Nd precipitates, that grow along 〈11
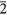
0〉_Mg_ in bulk *hcp*-Mg and on dislocation lines. The growth kinetics of β_1_ is sluggish even at high temperatures, and presumably occurs via vacancy migration. However, the rationale for the high-temperature stability of precipitate-matrix interfaces and observed growth direction is unknown, and may likely be related to the interfacial structure and excess energy. Therefore, we study two interfaces– {112}_β1_/{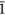
100}_Mg_ and {111}_β1_/{11
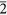
0}_Mg_– that are commensurate with β_1_/*hcp*-Mg orientation relationship via first principles calculations. We find that β_1_ acquires plate-like morphology to reduce small lattice strain via the formation of energetically favorable {112}_β1_/{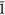
100}_Mg_ interfaces, and predict that β_1_ grows along 〈11
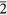
0〉_Mg_ on dislocation lines due to the migration of metastable {111}_β1_/{11
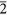
0}_Mg_. Furthermore, electronic charge distribution of the two interfaces studied here indicated that interfacial-energy of coherent precipitates is sensitive to the population of distorted lattice sites, and their spatial extent in the vicinity of interfaces. Our results have implications for alloy design as they suggest that formation of β_1_-like precipitates in the *hcp*-Mg matrix will require well-bonded coherent interface along precipitate broad-faces, while simultaneously destabilizing other interfaces.

Magnesium (Mg) alloys have tremendous potential as structural materials for automotive applications in engine block, transmission parts due to their lightweight. The concomitant fuel savings is however, thwarted by their unfavorable creep strength^1^,^2^,^3^,^4^. Mg-rare earth (RE) alloys are exceptions with demonstrated excellent creep resistance^1^,^2^,^3^. Their superior creep properties are correlated to the formation of high volume fractions of strengthening precipitates based on RE intermetallic compounds^4^,^5^. However, the scarcity and high cost of RE elements limits the development of creep–resistant Mg-RE alloys. This work is motivated by gaining an atomistic understanding of commonly found strengthening precipitates in creep-resistant Mg-RE alloys, and inform Mg-alloy design approaches by providing energetics and bonding characteristics associated with their presence within the *hcp*-Mg matrix. Therefore, we have investigated the structure of interface between intermetallic precipitate phase β_1_ (an ordered cubic structure) and *hcp*-Mg. This precipitate typically forms in Mg-Nd-based alloys like Mg-Nd, Mg-Nd-Y and Mg-Nd-Y-Zr, e.g. commercial WE43 and WE54 5,6,7. The coherent β_1_ phase has a plate-like morphology, and shares an (0001)_Mg_//(011)_β1_, [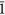
100]_Mg_//[21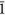]_β1_ and [11
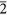
0]_Mg_//[1
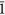
1]_β1_ orientation relationship (OR) with *hcp*-Mg^5^,^6^,^7^,^8^,^9^,^10^,^11^,^12^. TEM results also indicate that plate-like β_1_ precipitates always forms with a high aspect ratio, and its broad-face {21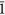}_β1_ parallel to {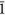
100}_Mg _4,5,6,7,8,9,10,11. Furthermore, β_1_ nucleating within the Mg-matrix is associated with the orthorhombic β′ precipitate phase^5^,^7^, or as self-accommodating β_1_ triads^5^,^11^, and heterogeneously along dislocation lines via accommodation of β_1_ stress-free transformation strains associated with β_1_ formation^7^,^8^,^9^,^13^,^14^. Heterogeneous formation is particularly useful in restraining high-temperature creep deformation, because its dynamic nucleation arrests dislocation movement and enhances the creep-resistance of Mg-RE alloys^4^,^9^,^10^,^11^.

The formation tendencies of β_1_ precipitates have been characterized experimentally^5^,^6^,^7^,^8^,^9^,^10^,^11^, and its evolution within Mg matrix and on dislocations studied via phase-field models^12^,^13^,^14^. Despite extensive studies on β_1_ phase, basic thermodynamic quantities like β_1_/Mg interfacial energies are yet to be determined unequivocally. Quantitative understanding of β_1_/Mg interfaces is crucial for three reasons. First, atomic resolution transmission electron microscopy (TEM) studies have shown that the{112}_β1_/{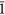
100}_Mg_ interfaces form both longer and shorter edges of β_1_ plates^11^. This is surprising. One would have expected at least an additional {111}_β1_/{11
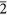
0}_Mg_ interface, because both {112}_β1_ and {111}_β1_ planes take part in forming the *hcp*-Mg/β_1_ orientation relationship. Second, a well-developed β_1_ plate shares a substantial interfacial-area with the parent Mg-matrix. Thus the interfacial energy, in conjunction with transformation strain energy, will likely determine the plate-like morphology^12^,^13^,^14^,^15^. Therefore, determination of *hcp*-Mg/β_1_ interfacial energies will provide valuable inputs to refine phase field models^12^,^13^,^14^,^15^. Third, this study allows us to rationalize whether these coherent interfaces have lower excess energies^16^.

Density functional theory (DFT)-based first principles calculations have been used in the past to determine interfacial energies of coherent precipitates in Mg-RE alloys^14^,^15^,^17^. So far, DFT studies on Mg-Nd have largely examined the stability of the β″ (ordered *hcp* structure^5^) and the β′ precipitate phases^15^,^17^; both precipitate phases form at early stages prior to β_1_ precipitation. For example, Issa *et al*. reported that β″ have positive and negative interfacial energies, when it joins with basal {0001}_Mg_ and prismatic {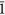
100}_Mg_ planes respectively^15^,^17^. Based on these interfacial energy calculations they proposed that β″ is unstable in Mg-Nd^17^, which was later corroborated with TEM observations^18^. The good agreement with experiments validates the available RE pseudopotentials with frozen-core f-electrons, and permits us to study other systems. However, the methodology used for determining negative interfacial energies needs further investigation because one intuitively associates an interface with positive excess energy. Therefore, the rationale for predicted negative interfacial energy was also investigated from the perspective of β_1_/Mg interfaces, especially since β_1_ is a key-strengthening phase in many commercial alloys^17^.

Previous DFT studies on Mg-Nd have not investigated β_1_ formation, its interfacial structure, and the associated bonding environment^14^,^15^,^17^. Furthermore, they did not examine thermodynamic quantities like surface excess energies - a difficult quantity to measure experimentally in the larger system limit^14^,^15^,^17^. Thus, guided by TEM studies^5^,^6^,^7^,^8^,^9^,^10^,^11^, we have investigated the energetics and structure of two types of β_1_/Mg interfaces, namely {112}_β1_/{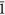
100}_Mg_ and {111}_β1_/{11
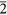
0}_Mg_, via DFT calculations.

## Computational Methodology

First principles computations were carried out with the Vienna Ab-initio Simulation Package (VASP) employing the projector-augmented plane-wave (PAW) method^18^, with electron exchange and correlation described by the generalized gradient approximation (GGA)^19^,^20^. All calculations were performed at 0 K, with a cut-off energy of 360 eV, k-point spacing of 0.1 Å^−1^, 0.2 eV Methfessel-Paxton smearing width and Brillouin zone integration at 10^−7^ eV convergence threshold. Groundstate structures of supercells were obtained by relaxing ionic, shape, and volumetric degrees of freedom till global energy convergence was achieved to within 1 meV/atom, and the Hellmann-Feynman forces on the atoms were less than 1 meV/Å. Visualization and subsequent analysis of the relaxed structures was performed using Vesta^21^ and Ovito^22^.

## Results

### Construction of initial supercells

**Figure 1:**
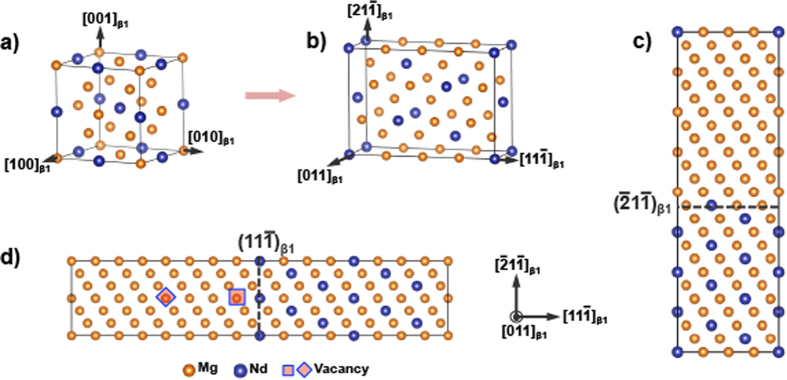
Crystal structures of (**a**) cubic β_1_ and (**b**) β_1_ in an orthorhombic cell. Pre-relaxed supercells extended along: (**c**) 〈112〉_β1_ and without any prior vacancies, and (**d**) 〈111〉_β1_ and showing the locations of vacancy placed within the “bcc” Mg-region.

Figure 1a shows β_1_ Mg3Nd with a BiF_3_ structure, lattice parameter 7.391 Å, space group Fm
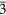
m (#226, similar to *fcc* structures), and Pearson symbol cF16 (corresponding to *bcc*-ordered D0_3_ *strukturbericht*)^23^. We constructed an orthorhombic β_1_ supercell whose edges are parallel to 〈011〉_β1_, 〈111〉_β1_ and 〈112〉_β1_. This choice corresponds to β_1_ - *hcp*-Mg orientation relationship seen in experiments^5^,^6^,^7^,^8^,^9^,^10^,^11^. Two orthogonal supercells containing 96 atoms, and lengths 35 (Fig. 1c) and 50 Å (Fig. 1d) were used in our analysis. In the resulting supercells (shown in Fig. 1c and d) 12 Nd atoms were replaced with Mg in one portion to create a “Mg-slab”, while retaining enough Nd atoms in the remainder to maintain Mg_3_Nd stoichiometry corresponding to β_1_. The resulting structures created {112}_β1_ (hereafter referred to as “{112}” supercell) and {111}_β1_ interfaces between Mg and the β_1_ precipitate as shown in Fig. 1c and d respectively. Note that in these initial supercells, Mg matrix has a “*bcc”* structure.

The Mg-slab was further modified based on the β_1_/Mg interfacial structure proposed by Nie and Muddle^5^. They suggested that β_1_ formation is associated with vacancies near the β_1_/Mg interface along 〈111〉_β1_ 5. Concomitantly, recent analytical calculations, involving stress-free transformations strains during β_1_ formation^11^,^13^, indicated the presence of a tensile strain along 〈111〉_β1_. Such tensile strains likely facilitate vacancy creation along 〈111〉_β1_ by expanding the volume near the interfaces. Therefore, to understand such effects a Mg vacancy was introduced near the β_1_/Mg interface (square, Fig. 1d) and at the center of Mg (diamond, Fig. 1d) in separate supercells. An Mg vacancy is placed inside “Mg” slab, rather than in the intermetallic β_1_ compound, was guided by β_1_/Mg interfacial structure proposed in literature^6^,^11^. Also, this reasonable because Mg vacancy formation energies in β_1_ (1.24 eV) is greater than in *hcp*-Mg (0.82 eV). The resulting vacancy concentration was ~1 at% for the entire supercell, and 2at% for the Mg slab. Hereon the vacancy containing supercells - near β_1_/Mg interface, and center of Mg slab - will be referred to as “{111}_β1(Iv)_” and “{111}_β1(Cv)_” supercells respectively.

### Relaxed structures

**Figure 2:**
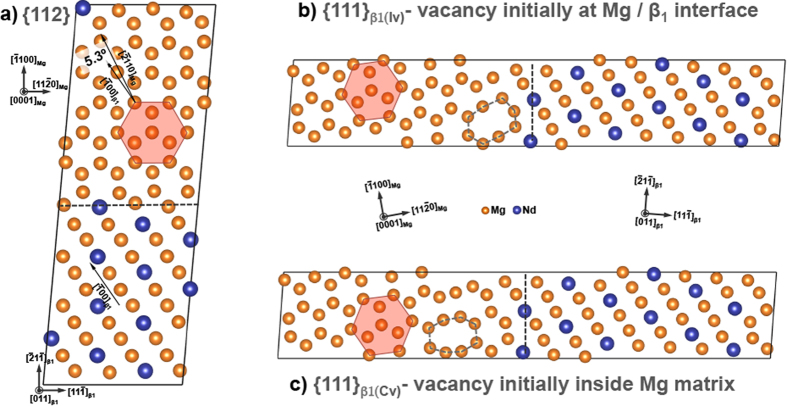
Relaxed supercells showing β_1_/Mg interfaces. (**a**) {112}_β1_/{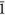
100}_Mg_ interface, (**b**) {111}_β1(Iv)_/{11
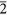
0}_Mg_ interface, with a vacancy that was initially placed near the interface, and (**c**) {111}_β1(Cv)_/{11
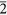
0}_Mg_ interface with a vacancy was initially placed at the center of Mg-region.

The relaxed structures of three supercells with β_1_/Mg interfaces are shown in Fig. 2. In the {112}, {111}_β1(Iv)_ and {111}_β1(Cv)_ supercells (Figs 1a and 2b,c respectively), the Mg slab with an initial *bcc* structure transforms to the correct *hcp* crystal structure indicated by hexagons in Fig. 2. The β_1_ however, retains its *bcc*-ordered structure as confirmed by common neighbor analysis (CNA)^22^,^24^. In this manner we created “equilibrium” supercells comprising of two types of β_1_/*hcp-*Mg interfaces: {112}_β1_//{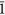
100}_Mg_ and {111}_β1(Iv/Cv)_//{11
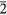
0}_Mg_. The structural relaxation of {111}_β1(Iv)_ and {111}_β1(Cv)_ supercells (Fig. 2b and c), with an added vacancy (Fig. 1d), also created a region with vacancy-like excess free volume marked by dotted polygons in Fig. 2b and c. This occurs even when a single vacancy was initially placed at the center of the Mg slab (Fig. 1d). The excess volume of *hcp-*Mg in Fig. 2b and c measured by Voronoi tessellation^21^, was ~30 Å^3^, and ~23 Å^3^. In comparison, Mg slabs without prior vacancies inside the {111} supercell, did not have an excess volume regions and neither did their initial *bcc* Mg relax to a *hcp* structure (Supplementary Figure S1) – unlike those presented in Fig. 2b and c. Thus vacancies aid in the transformation of *bcc*-Mg to *hcp*.

In the relaxed {112} supercell, Fig. 2a, certain crystallographic orientations of Mg and β_1_ were mutually coincident and exhibited the following orientation relationship (OR): (0001)_Mg_//(011)_β1_, [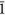
100]_Mg_//[21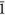]_β1_, and [11
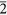
0]_Mg_//[1
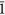
1]_β1_. Electron diffraction patterns from literature have indicated that the β_1_-Mg OR causes a ~5.26° misorientation between (0001)_Mg_ and (011)_β1_ planes^5^,^6^,^7^,^8^,^9^,^10^,^11^, which is in excellent agreement with DFT value of 5.3° indicated in Fig. 2a, and, also validates our approach. The misorientation can be quantified as the angle between [1
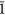
00]_β1_ and [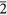
110]_Mg_.

A comparison of β_1_ and Mg crystallographic directions within {111}_β1(Iv)_ and {111}_β1(Cv)_ supercells showed that, eventhough (0001)_Mg_ and (011)_β1_ planes are parallel, the other crystallographic axes involved in β_1_-Mg OR are not coincident. For example, angle between [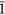
100]_Mg_ and [21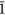]_β1_ in {111}_Iv_ and {111}_Cv_ is ~15°, while those axes are coincident in case of the {112} supercell. In other words, DFT calculations suggest that a creation of only {111}_β1(Iv/Cv)_/{11
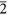
0}_Mg_ interfaces may not yield the experimentally seen β_1_-Mg OR atleast near the interfacial regions. This result is surprising because, based on the β_1_-Mg orientation relationship, one would have expected a mutual alignment of β_1_ and Mg orientations in the {111}_β1(Iv/C/v)_ supercells.

Implication of this “off β_1_-Mg OR” misalignment in the {111}_β1(Iv/C/v)_ supercells was further probed by comparing it’s local atomic distortions with the {112} supercells. Mg-Mg, Mg-Nd and Nd-Nd bond-lengths measured from regions 3–4 atomic layers away from the interfaces were used to calculate the lattice strain 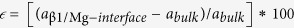, where *a*_β1/Mg-*interface*_ and *a*_*bulk*_ are the lattice parameters of each phase in the supercells, and their bulk equilibrium lattice respectively. Table 1 lists the β_1_ and Mg lattice parameters, and the lattice strains. Lattice strains, 5–10 times larger that the bulk systems, were found in {111}_β1(Iv)_ and {111}_β1(Cv)_ supercells along the a-axis of Mg or 〈11
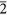
0〉 and 〈100〉 of β_1_. We further note that placement of vacancy away from {111}_β1(Iv)_/{11
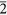
0}_Mg_ interface (Fig. 1d) increases the β_1_ lattice strain by ~33% in {111}_β1(Cv)_ in comparison to {111}_β1(Iv)_. In other words, higher lattice strains are associated with the formation of {111}_β1_/{11
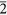
0}_Mg_ interfaces than {112}_β1_/{01
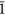
0}_Mg_. Regardless, presence of vacancy-like excess volume in the {111}_β1(Iv/Cv)_ supercells strains the Mg and β_1_ lattice, and will be shown to affect the β_1_/Mg interfacial energies.

### β_1_/Mg interfacial energies

**Figure 3:**
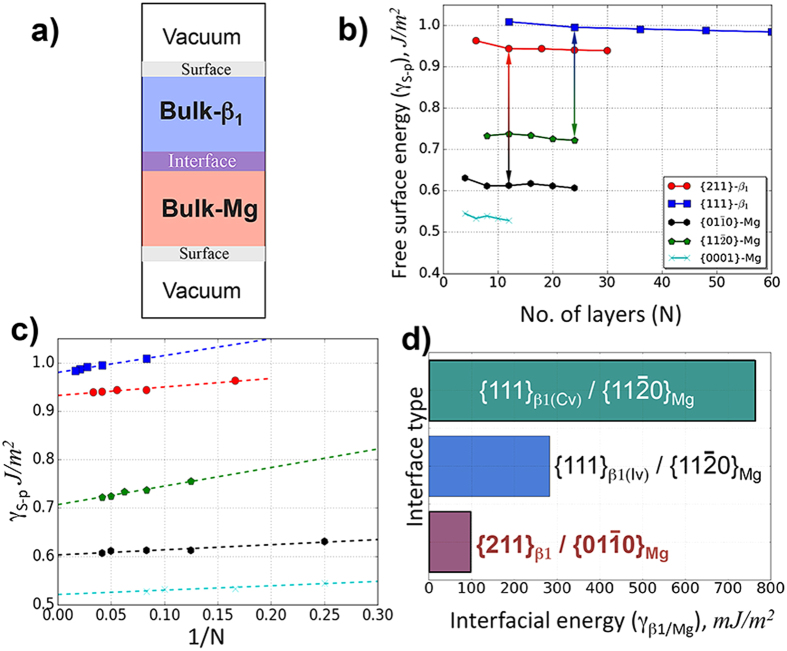
(**a**) Configuration used for calculating β_1_/*hcp-*Mg interfacial energy. (**b**) Free surface energies (γ_S-p_) of different β_1_ and Mg surface planes plotted as a function of number of atomic layers (N). (**c**) γ_S-p_ vs 1/N plots to determine γ_S-p_ in the larger size limit. The dotted line fits corresponding to R^2^ values of 0.94. The intercept of the linear fits, 1/N → 0 yield excess surface energy in the large system limit (N → ∞), which is a well-defined thermodynamic quantity. (**d**) Bar chart showing the interfacial energies of three *β*_1_/Mg interfaces reveal {112}_β1_/{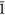
100}_Mg_ interfaces are energetically more stable than {111}_β1_/{11
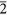
0}_Mg_.

The geometry used for calculating β_1_/Mg interfacial energies (γ_β1/Mg_) is schematically shown in Fig. 3a as vacuum-space/bulk-β_1_/bulk-Mg/vacuum-space. Here the interacting β_1_/Mg surfaces are {111}_β1(Iv/Cv)_/{11
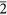
0}_Mg_ and {112}_β1_/{01
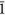
0}_Mg_ (see Supplementary Figure 2). This methodology was previously used by Liu *et al*. to examine the interface between an ordered intermetallic compound and the disordered matrix^25^. This approach further avoids the development of excess artificial interfacial strains by having vacuum on both sides of the unconstrained interfacial supercell. Thus, one need not include additional coherency strain energy terms (e.g. ref. 17) to evaluate the interfacial energies. Furthermore, since β_1_ and Mg are exposed to the vacuum on either ends (Fig. 3a), we are required to determine the “free” surface of the participating {111}_β1,_ {11
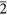
0}_Mg_, {112}_β1_ and {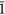
100}_Mg_ planes. The free surface energy, γ_S-p_, for a phase “p” was calculated using the expression 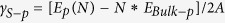, where E_p_(N) is the calculated ground state energy for a “N” atomic layer supercell slab of phase “p”, E_Bulk-P_ is the bulk energy of phase “p”, and A is the surface area^25^,^26^,^27^. The calculations involved a maximum of 24 (~35 Å) and 60 (~50 Å) layers for Mg and β_1_ respectively, and a minimum of ~12 Å of vacuum space. E_Bulk-P_ was determined from the slope of E_p_(N) vs N plot. For pure metals, E_Bulk-P_ corresponds to their cohesive energy^27^, and our calculations yielded 1.53 eV/atom for Mg - in excellent agreement with literature^26^,^27^,^28^. Figure 3b shows that plot of γ_S-p_ vs. N for different β_1_ and Mg planes, and the results indicated that the γ_S-p_ values, for {111}_β1(Iv/Cv),_ {11
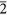
0}_Mg_, {112}_β1_ and {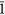
100}_Mg_ planes rapidly converge within the chosen range of N.

The γ_S-p_ values for the Mg and β_1_ planes were calculated using linear fits (R^2^ ≈ 0.94) to γ_S-p_ vs. 1/N plots (Fig. 3c). We then extrapolated the fits for 1/N → 0, which correspond to N → ∞ layers or the “bulk”. Our surface energy γ_S-p_ of (0001)_Mg_ was 0.52 J/m^2^, agreed well with literature reports of 0.52 and 0.55 J/m^2^ 26,27. Figure 3b shows that β_1_ has higher excess surface energies (γ_S-p_) than pure Mg. This indicates a stronger bonding within β_1_, and that it is difficult to cleave or fracture β_1_ during deformation. We further note that such surface excess energies have not been reported for any other precipitate phases in Mg-RE systems^14^,^15^,^16^.

The plots in Fig. 3b and c helps estimate the optimal value of “N” required for the interfacial energy calculations that reasonably accounts for the bulk phases along with the free-surface and interfacial energies. Thus, 12 (~15 Å) and 24 (~25 Å) layers was chosen for each phase making up the {112}_β1_/{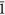
100}_Mg_ and {111}_β1_/{11
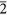
0}_Mg_ interfaces respectively, and ~12 Å of vacuum was maintained above β_1_ and Mg slabs (Fig. 3a). β_1_/Mg interfacial energy (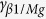) was estimated using the expression^22^:


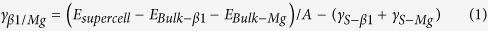


where γ_S_-_β1_ and γ_S_-_Mg_ are the free surface energies of β_1_ and Mg respectively, E_supercell_ is the ground state energy of the β_1_-Mg supercell while E_Bulk-β1_ and E_Bulk-Mg_ are the bulk energies of β_1_ and Mg respectively, and A is the surface area. The γ_β1/Mg_ values of these three interfaces are plotted in Fig. 3d as a bar chart. Their respective relaxed structures are also shown in Supplementary Figure 2. The calculated γ_β1/Mg_ values for {112}_β1_, {111}_β1(Cv)_ and {111}_β1(Iv)_ supercells were 98, 282 and 762 mJ/m^2^ respectively. Thus, the {112}_β1_/{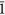
100}_Mg_ interfaces are more energetically stable than {111}_β1_/{11
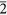
0}_Mg_. Also note that inclusion of excess surfaces energies for estimating interfacial energies (equation 1) will result in only positive excess energy values, which is thermodynamically reasonable.

### Structure and bonding environment near β_1_/Mg interfaces

**Figure 4:**
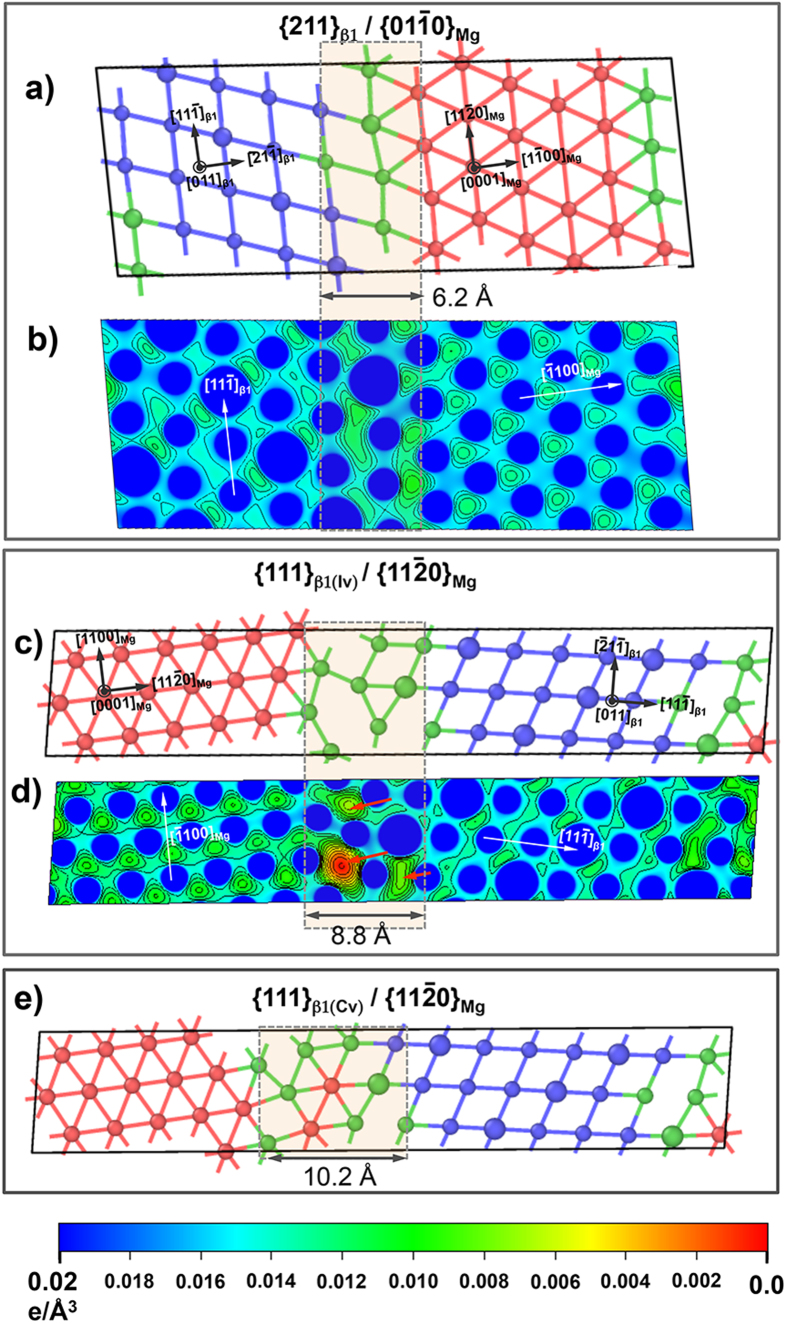
(**a,b**) and (**c,d**) show structure and electron charge distributions of {112}_β1_/{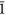
100}_Mg_ and {111}_β1(Iv)_/{11
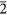
0}_Mg_ interfaces respectively. In (**a**) and (**b**) red and blue colors indicate atomic sites in *hcp*-Mg and β_1_ structures respectively, while green indicates “distorted” lattice sites. (**e**) shows the legend for electron charge densities in (**b**) and (**d**). (**e**) shows the structure of{111}_β1(Cv)_/{11
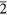
0}_Mg_ interface. Interfacial width is indicated by translucent boxes, and the contours in the electron charge density plots engulf regions with lower charge densities compared to the bulk.

To explain the lower interfacial energy of {112}_β1_/{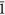
100}_Mg_ in Fig. 3d, we have compared the bonding environment of these interfaces. The analysis correlates the interfacial atomic-coordination (Fig. 4a and c), with electron charge density distribution (Fig. 4b and d).

In Fig. 4a and c the atom colors are assigned by CNA environment type^18^,^20^ and the bonds depict in-plane – i.e. (0001)_Mg_//(011)_β1_ – coordination around an atomic site: (i) red denotes *hcp-*Mg with 6 in-plane coordination, (ii) blue denotes *bcc*-ordered β_1_ with 4 in-plane coordination, and (iii) green depicts sites which were not identified as either *hcp-*Mg or β_1_. Thus, the green-colored bonds/atoms near the interfacial regions - indicated with shaded boxes across Fig. 4a–d - can be interpreted as distortion of the lattice sites, from bulk *hcp-*Mg and β_1_ structures.

A comparison of the interfacial regions, and their corresponding bonding environment reveal that the distorted lattice sites (shown by green colored bonds) are invariably located around regions with significant depletion of electron charge density distributions (marked by red arrows in Fig. 4b and d). In other words, interfacial lattice distortions correlate well with lower electron density or weak bonding^29^. Furthermore, the electron density distributions near the interfacial regions of {111}_β1(Iv)_/{11
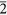
0}_Mg_ was discernibly lower (compare Fig. 4b and c with legend at the bottom), and spread over a wider region than {112}_β1_/{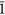
100}_Mg_. Correspondingly, lattice significantly distorted near the {111}_β1(Iv)_/{11
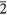
0}_Mg_ interface. Therefore, considering the larger differences in the energies of {112}_β1_/{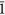
100}_Mg_ (98 mJ/m^2^) and {111}_β1(Iv)_/{11
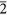
0}_Mg_ (282 mJ/m^2^) interfaces, it is evident that smaller population of distorted lattice sites and narrow spatial extent near the interface correlate with lower interfacial energy. This is further confirmed for {111}_β1(Cv)_/{11
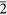
0}_Mg_ system (762 mJ/m^2^) in Fig. 4e, which displayed a wider spatial extent of distorted interfacial lattice sites (overlapping both *hcp-*Mg and β_1_ structures) than the other two interfaces.

## Discussion

One of our motivations was to rationalize the existence of only {112}_β1_/{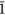
100}_Mg_ interfaces seen in Mg-Nd alloys, which was contrary to the exceptions based on β_1_/Mg OR^11^. The DFT calculations revealed that, unlike {112}_β1_/{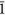
100}_Mg_, formation of {111}_β1_/{11
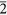
0}_Mg_ requires the presence of vacancy-like excess volume near the interfaces, which causes interfacial distortions, higher lattice strains and presumably strain energy within the mating phases. Such pronounced distortion/strain increases the interatomic spacings near the interface, reduces charge density between the atoms, and creates weaker interfacial bonding in {111}_β1_/{11
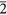
0}_Mg_. The relatively poor bonding increases the excess energy of {111}_β1_/{11
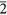
0}_Mg_ incomparison to the {112}_β1_/{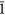
100}_Mg_ interface, and makes the latter an energetically preferable interface.”

Despite the fact that excess volume results in an energetically unstable {111}_β1_/{11
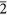
0}_Mg_ interface. However, the presence of such excess volume near the interfaces may explain the growth of plate-like β_1_ within Mg-matrix and along dislocation lines^5^,^6^,^7^,^8^,^9^,^10^,^11^. Nie and Muddle have proposed that shear transformation strains are associated with β_1_ formation, because of which excess vacancy concentration is created at the short ends oriented along 〈11
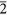
0〉_Mg_. It is likely β_1_ growth can occur via vacancy migration to the interfaces. However, to maintain elongated plate-like morphology during the growth process, β_1_ needs a stable interface in its long edge. Our DFT results suggest that a low lattice strain and interfacial energy of {112}_β1_/{100}_Mg_ interfaces allows β_1_ precipitates to acquire a plate-like morphology with a high aspect ratio and possess a broad {112}_β1_-face parallel to {1
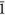
00}_Mg_ as seen in experiments^4^,^5^,^6^,^7^,^8^,^9^,^10^,^11^,^12^. In the case of dislocation assisted β_1_ form as linear chains along 〈11
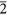
0〉_Mg_ 10,13,30, which are also observed in our creep-tested microstructures^10^. We postulate that such linear growth of β_1_ along dislocation lines occur via transfer of vacancies to metastable interfaces like {111}_β1(Iv)_/{11
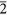
0}_Mg_ (Fig. 2b), which tends to have lower interfacial energy than vacancy formation away form the β_1_/Mg interface (i.e. {111}_β1(Cv)_/{11
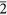
0}_Mg_ in Figs 2c and 3d). Since dislocations can rapidly supply vacancies via dislocation pipe diffusion, the diffusion assisted movement of {111}_β1(Iv)_/{11
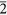
0}_Mg_ type interfaces rapidly consumes a large portion of the host dislocation line, and orients β_1_ along 〈11
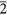
0〉_Mg_. Finally, since {111}_β1(Iv)_/{11
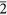
0}_Mg_ interfaces are energetically unfavorable, interfacial atomic rearrangements, e.g. via shear process^5^,^6^, produces {112}_β1_/{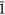
100}_Mg_ interfaces in the final microstructure.

From the perspective of alloy design approaches; the contribution of this report is two fold. We demonstrate that in order to develop alloys with advantages associated with “β_1_-like” precipitation one needs to (i) retain a well bonded coherent interface at the precipitate broad faces, e.g. {112}_β1_/{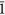
100}_Mg_, while simultaneously (ii) form mobile yet unstable interfaces on the short edges to allow precipitate growth along dislocation lines. However, finding non-RE substitutions, which fits these two criteria, is our next challenge, and is currently being tackled by coupling evolutionary algorithms with first principle calculations.

## Conclusion

Formation of nanoscale β_1_-Mg_3_Nd precipitates in *hcp*-Mg matrix is known to enhance the creep resistance of Mg-alloys because of their tendency to form on dislocation lines and sluggish coarsening kinetics when they form in the *hcp* alloy matrix. Using first principles calculations, we have compared the excess energy and the structure of {112}_β1_/{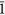
100}_Mg_ and {111}_β1_/{11
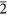
0}_Mg_ interfaces. Our calculations revealed that the formation of {112}_β1_/{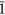
100}_Mg_ interfaces, compared to {111}_β1_/{11
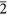
0}_Mg_ interface, is associated with significant reduction in both the interfacial energy and lattice strains in the adjacent β_1_ and Mg matrix. The favorable formation energetics of the {112}_β1_/{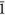
100}_Mg_ interface, in conjunction with small lattice strains, influences β_1_ acquiring a plate-like morphology with its broad-face {112}_β1_ parallel to {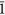
100}_Mg_. Electronic structure of these interfaces revealed that a lower interfacial energy also correlates with a smaller population of distorted lattice sites near the interfacial regions. Additionally, our DFT investigation informs that creep-resistant Mg alloys will benefit from “β_1_-like” precipitation because of two key features (i) retain a well bonded coherent interface at the precipitate broad faces, e.g. {112}_β1_/{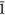
100}_Mg_, and (ii) destabilize other interfaces (corresponding to precipitate-Mg OR) to promote growth along a desired direction, e.g 〈11
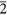
0〉_Mg_.

## Additional Information

**How to cite this article:** Choudhuri, D. *et al*. Interfacial structures and energetics of the strengthening precipitate phase in creep-resistant Mg-Nd-based alloys. *Sci. Rep.* **7**, 40540; doi: 10.1038/srep40540 (2017).

**Publisher's note:** Springer Nature remains neutral with regard to jurisdictional claims in published maps and institutional affiliations.

## Supplementary Material

Supplementary Materials

## Figures and Tables

**Table 1:** Table of lattice parameters and strain in Mg and β_1_ slabs of the relaxed structures.

Interface	Mg matrix		β_1_ precipitate a (Å)/strain (%)
	a (Å)/strain (%)	c(Å)/strain (%)	
{211}_β1_/{0110}_Mg_ – No vacancy	3.186/−0.28	5.206/+0.51	7.385/− 0.37
{111}_β1_/{1120}_Mg_ – Vacancy initially at the interface	3.282/+2.73	5.202/+0.43	7.282/−1.76
{111}_β1_/{1120}_Mg_ – Vacancy initially inside Mg	3.276/+2.53	5.220/+0.78	7.239/−2.34

Calculated lattice parameters of bulk phases: Mg − 3.195 Å and 5.180 Å and β_1_ − 7.412 Å.
