# Supplementary material for: Interfacial structures and energetics of the strengthening precipitate phase in creep-resistant Mg-Nd-based alloys
Source: Sci Rep. 2017 Jan 17;7:40540. doi: 10.1038/srep40540 (PMC5240141; doi:10.1038/srep40540)
Supplement: Supplementary Materials [file srep40540-s1.pdf]

**Supplementary Materials**

# Interfacial structures and energetics of the strengthening precipitate phase in creep-resistant Mg-Nd-based alloys

D. Choudhuri[*], R. Banerjee[$] and S.G. Srinivasan[#]
Department of Materials Science and Engineering, University of North Texas, Denton, TX, 76191, USA

**Correspondence:** [*]deep.choudhuri@gmail.com; [$]raj.banerjee@unt.edu; [#]srinivasan.srivilliputhur@unt.edu

**Supplementary Figure -1**

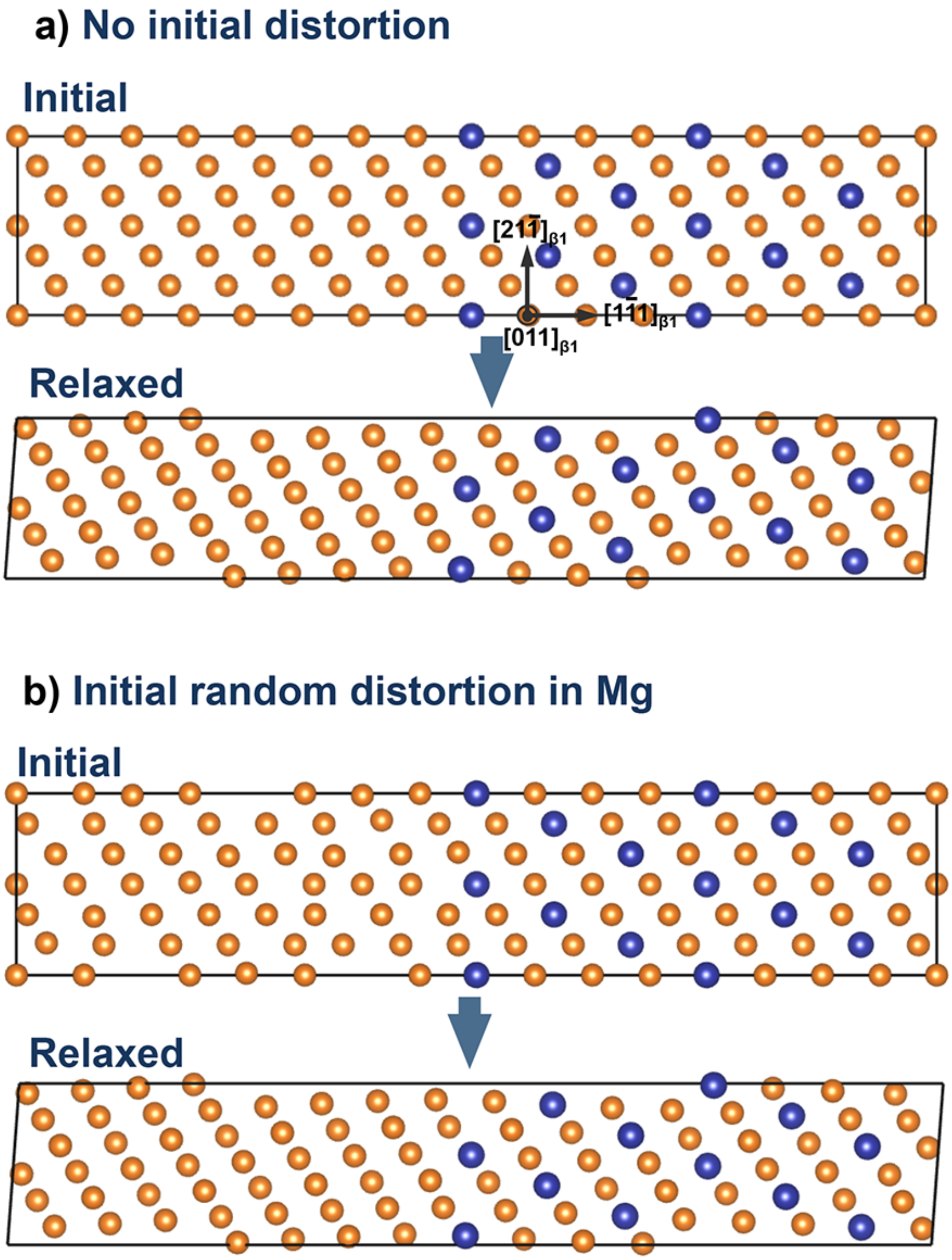


**Supplementary Figure 1:** Initial and relaxed (energy minimized) structures of supercells along <111>$\beta_1$ : (a) no initial distortion were imposed in Mg side, and (b) (0.1 – 1Å ) random distortions imposed on Mg atoms in the Mg side.

**Supplementary Figure -2**

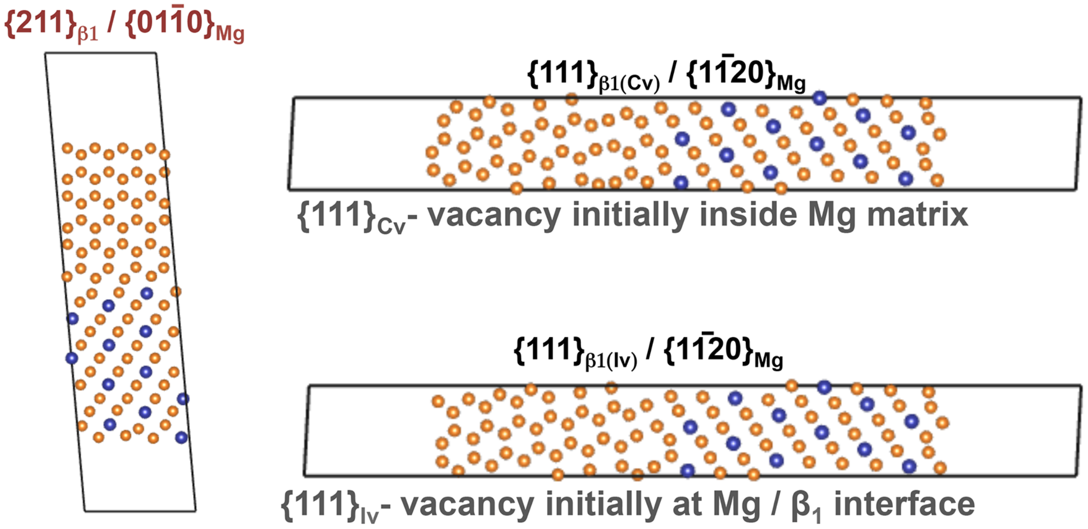


**Supplementary Figure 2:** Relaxed interfacial structures with configuration vacuum-space / bulk-$\beta_1$ / bulk-Mg / vacuum-space..
